# Topical Gel of Vitamin A Solid Lipid Nanoparticles: A Hopeful Promise as a Dermal Delivery System

**DOI:** 10.34172/apb.2021.075

**Published:** 2020-10-03

**Authors:** Mahshid Boskabadi, Majid Saeedi, Jafar Akbari, Katayoun Morteza-Semnani, Seyyed Mohammad Hassan Hashemi, Amirhossein Babaei

**Affiliations:** ^1^Student Research Committee, Faculty of Pharmacy, Mazandaran University of Medical Sciences, Sari, Iran.; ^2^Department of Pharmaceutics, Faculty of Pharmacy, Mazandaran University of Medical Sciences, Sari, Iran.; ^3^Department of Medicinal Chemistry, Faculty of Pharmacy, Mazandaran University of Medical Sciences, Sari, Iran.

**Keywords:** Vitamin A, Solid lipid nanoparticle, Dermal delivery, Skin irritation

## Abstract

***Purpose:*** The Objective of the present investigation was to enhance the skin delivery of vitamin A (Vit A) via producing solid lipid nanoparticles (SLNs) through ultrasonication technique.

***Methods:*** For achieving optimal skin delivery, impacts of two surfactants ratio of Tween80:Span80 on nanoparticles (NPs) features and the respective functions were examined. Powder X-ray diffractometer (PXRD), photon correlation spectroscopy, attenuated total reflectance-Fourier transform infrared spectroscopy (ATR-FTIR), transmission electron microscopy (TEM), and differential scanning calorimetry (DSC) were applied for characterizing the solid state of Vit A in the SLN.

***Results:*** Results showed that size of the NPs is usually enhanced by adding co-emulsifier (Span80). Notably, minimum NPs size (64.85±4.259 nm) was achieved while the hydrophilic-lipophilic balance (HLB) of the binary surfactants was 12.08, close to HLB of beeswax (HLB=12) as lipid matrix. Also, maximum entrapment efficiency (66.01±8.670%) was observed in the formulation. DSC thermogram indicated an amorphous form of Vit A in SLN. ATR-FTIR spectra of Vit A-SLN illustrated that prominent functional groups are found in the formulations that might be a sign of acceptable entrapment of Vit A in a lipid matrix. Moreover, ATR-FTIR studies showed no chemical interactions between Vit A and excipients. Skin irritation test proved the non-irritancy of Vit A-SLN2, when applied to the dorsal region of Wistar rats. Finally, any cellular toxicity was not seen for NPs.

***Conclusion:*** It was found that the procured Vit A-SLNs could be utilized as potent carriers for the dermal delivery of Vit A.

## Introduction

One of the fat-soluble vitamins involving in forming and maintaining healthy skin, hair, and mucous membrane is vitamin A (Vit A). Vit A plays a role in increasing skin elasticity, reducing roughness, and preventing peroxidation of skin lipids via exfoliation of the skin top layer that speeds up cell renovation, and make the skin a fresher, more uniform, and younger appearance. Moreover, its roles as an anti-oxidant and prevents tissue atrophy and collagen loss during aging.^1^ Due to its anti-oxidant and moisturizing features, Vit A is usually observed in creams or gels for topical use. Nevertheless, it has high sensitivity to ultra-violet radiation and oxygen that may quickly enhance its degradation, which result in declining the contents of Vit A in epidermis.^2^ Hence, a proper drug delivery system is needed for preserving this lipophilic compound activity. For resolving such problems, approaches including conservation of materials for maintaining stability and natural features for a lengthy shelf-life would be interesting. Researchers investigated several encapsulation methods for increasing retinyl acetate stability against such potent degrading parameters.^3^ Using solid lipid nanoparticles (SLNs) is one of the above-mentioned methods.

SLNs were designed in the early 1990s as a substitute carrier system for liposomes, emulsions, and polymeric nanoparticles (NPs).^4^ Researchers claimed that SLNs incorporate the benefits and prevent disadvantages of other colloidal carriers.^5^ The suggested benefits are possibly controlled drug release and drug targeting, lack of biotoxicity of the carrier and of concerns with regard to large scale manufacturing, sterilization, as well as possible increase of drug stability. Therefore, SLNs were recommended for widespread utilization such as parenteral,^6^ oral,^7^ and dermal^8-10^ routes. *Stratum corneum* is a key obstacle for per-cutaneous absorption of topically administered drugs. Little and partially narrow size distributions of SLN provide location-specific delivery to the skin.^11^ SLNs possess high affinity with the stratum corneum, and thus provide a higher bio-availability of the encapsulated materials to the skin. They increase transportation and penetration of active materials, specially lipophilic agents, and concentration of the agents in the skin.^4^ Several research published using lipid NPs for dermal utilization of cosmetic and pharmaceutical active materials, including ascorbyl palmitate, tocopherol (vitamin E), co-enzyme Q10, and retinol (Vit A).^1^ Chemical stability of these sensitive actives can be augmented if they are combined into the drug carrier systems. Authors particularly examined the stabilization effects of SLN on retinol, and proposed dependence of stabilization on the kind of lipids and surfactants employed.

The hydrophilic-lipophilic balance (HLB) conception, is that the known technique to choose a chemical agent appropriate for associate application. This empirical technique assigns the chemical agent a HLB variety in line with its chemical structure. Many numerical as well as experimental strategies are introduced during the last years to work out HLB numbers. These strategies, ab initio developed for nonionic surfactants, are mainly based on the individual size of hydrophobic and deliquescent moiety of chemical agent molecules. HLB numbers permit the power of chemical agent to stabilize o/w or w/o emulsions for foreseen. The mentioned condition is often due to the conception of the ‘required HLB’ or ‘effective HLB value’. The specified HLB is that the chemical agent HLB, or a mix of surfactants, that permits achieving the foremost stable emulsion for a defined mechanism (processing condition, oil phase, and so on). For estimating the specified HLB, the emulsions were prepared with an equivalent binary compound and oily phases however totally different surfactants at intervals vary of HLB. Therefore, the specified HLB corresponded to the most stability of the emulsion. Vit A chose as a lipophilic agent for incorporating into SLNs via ultrasonication technique, and effects of the HLB of surfactant mixes on SLN features was investigated. Hence, in the current research, attempts were made to prepare the Vit A loaded SLN using ultrasonication methods. The properties of SLNs were also optimized by the role of HLB. The dermal penetration and deposition of Vit A-SLN through rat skin were studied to discover the potential use of SLN of Vit A in topical formulations.

## Materials and Methods

### Materials

Vit A was obtained from Osve Pharmaceutical Company (Tehran, Iran). Tween 80, Span 80, Ethanol, Methanol and tri-ethanolamine bought from Merck (Merck Company, Germany). Beeswax (BW) achieved from John’s Laboratory (John’s Laboratory Chemicals, India). Then, a Human power 2 system (human Co., Korea) used to purify the distilled water. Carbopol 941 selected from B.F Goodrich (B.F Goodrich Chemical Co., UK).

### Preparing Vit A-SLN

Vit A NPs procured via the probe ultrasonication technique.^12^ A mix of BW, span 80, and Vit A melted through a heater stirrer at below 70°C. Heating the surfactant solution (Tween 80 & water) accomplished to 75 to 80°C. The preheated surfactant solution combined with the heated mix of lipids and Vit A for forming a preemulsion via a hot plate magnetic stirrer. Sonication of the mix continued by a probe sonicator for two minutes (Bandelin, 3100, Germany). Then, it was directly submerged in an ice bath after finishing the sonication procedure. Table 1 shows the composition of investigated formulation.

**Table 1:** Component and physicochemical properties of investigated Vit A-SLN

**Formulation**	**Vit A**	**BW**	**Tween 80**	**Span 80**	**Water Up to**	**HLB**	**Particle size (nm)**	**PDI**	**ZP (mv)**	**EE%**
Vit A-SLN1	0.5%	2.5%	3.40%	0.03%	100%	14.90	197.67±22.942	0.278±0.019	-2.87±0.20	42.39±6.110
Vit A-SLN2	0.5%	2.5%	3.01%	0.38%	100%	13.80	275.00±29.860	0.321±0.015	-3.16±0.44	61.30±6.700
Vit A-SLN3	0.5%	2.5%	2.49%	0.93%	100%	12.08	64.85±4.259	0.292±0.035	-4.34±0.21	66.01±8.670
Vit A-SLN4	0.5%	2.5%	1.99%	1.44%	100%	10.05	348.67±14.572	0.416±0.017	-5.41±0.30	54.42±9.171
Vit A-SLN5	0.5%	2.5%	1.38%	2.05%	100%	8.70	272.67±5.131	0.475±0.010	-6.63±0.36	14.50±2.681
Vit A-SLN6	0.5%	2.5%	0.77%	2.66%	100%	6.90	486.00±8.888	0.547±0.019	-0.65±0.19	58.23±2.067

### Preparing Vit A-SLN gel and Vit A suspension gel

For preparing the plain gel, carbopol (1.5%) was dispersed in preserved water and maintained overnight. Then triethanolamine was used to neutralize the carbopol solution. For preparing Vit A-SLN gel, 70 g of Vit A nano-dispersion (0.5% Vit A) was combined with 30 g simple gel based on the propeller homogenizer at 400 rpm. In addition, for preparing Vit A suspension gel, Vit A suspension (0.5% Vit A) was blended with 30 g simple gel under the propeller homogenizer at 400 rpm.^13^

### Rheological characterization of Vit A-SLN gel

The viscosity of prepared Vit A-SLN gel was examined using S-94 spindle on the Brookfield viscometer (DV-II Pro Viscometer, Middleboro, MA). A continuous variation of the speed scale from 0.5 to 100 rpm (25 ± 1.0°C) was performed for sample, and the subsequent viscosity was measured.^14^

### Physico-chemical description

In this section, dynamic light scattering technique via a Zetasizer Nano ZS system (Malvern Instruments; Worcestershire, UK) with 90° angle at 25°C was used to determine average size of the particle, polydispersity index (PDI), and zeta potential (ZP) of Vit A-SLN.^15^

### Entrapment efficiency

The Vit A-SLN was exposed to centrifuging at 27 000 rpm for 90 minutes (Sigma, Germany) for separating the loaded Vit A from dispersion. Filtration of the supernatant was done via a syringe filter (pore size of 0.22 μm) and amounts of Vit A in supernatant (free drug) was specified via HPLC Knauer equipped with Knauer Eurospher 100-5 C18 column (5 μm, 4.6 × 250 mm).

Moreover, the mobile phase was a 100% methanol and flow rate was 1 mL/min. UV wavelength was detected at 325 nm. The drug retention lasted for seven minutes. Drug entrapment efficiency (EE%) was computed through Equation (1):

Eq. (1) $$EE\%=\frac{W_{initial}-W_{free}}{W_{initial}}\times 100$$

### Transmission electron microscopy

According to the research design, transmission electron microscopy (TEM) (Hitachi H-7500: Japan) was applied for morphological observations, which is operated at 120 kV. In brief, SLN formulation was initially diluted twice with the distilled water. A drop of diluted samples was inserted on a 200-mesh carbon coated copper grid that has been stained with 2% phosphor tungstic acid solution. Then, drying was done at the room temperature. Finally, the representative images of all samples were illustrated.

### ATR-FTIR spectroscopic analyses

Vit A–excipient interactions were examined via Cary 630 FTIR spectro-photometer (Agilent Technology Inc., CA; USA) with a diamond attenuated total reflectance (ATR) (the lessened total reflectance). Vit A, BW, tween 80, span 80 as well as the respective physical mixes were exposed to ATR-FTIR examination. With regard to this technique, recording the spectra was done via placing little amounts of all homogeneous samples on the window and scanning between wave number ranges 400-4000 cm^-1^ and 2 cm^-1^ of resolution.

### Differential scanning calorimetry analyses

Differential scanning calorimetry (DSC) was measured on the bulk ingredient as well as the Vit A-SLN powder through a Pyris 6 (PerkinElmer, USA). Weighing and sealing 5 mg of BW specimens, model drug as well as the freeze-dried SLN formulations was done in the aluminum pans. The sealed pans were maintained under isothermal condition at 20°C for 30 minutes. When it reached balance, DSC thermograms for bulk specimens, and Vit A-SLN powder was registered from 20-250°C at the heating rate of 20°C/min based on the inert nitrogen gas atmosphere.^15^ Indium was used to calibrate DSC prior to experimenting the specimens.

### PXRD analyses

Diffraction patterns were determined by X-ray diffractometer (PHILIPS-PW1730, the Netherland) (40 kV, 30 mA) for identifying all modifications in the crystal lattice of substances following generating SLNs. The crystalline features of Vit A, BW, and freeze dried SLN powder was examined via subjecting them to the Cu Kα radiation with 1.5406 Å wavelength. Then, scanning was performed from 10.000-80.000°, 2Ɵ at the step size equal to 0.050°, and finally the step time equal to one second.

### In vitroskin permeation examination

This research followed the Ethical Instructions for Examinations in the Laboratory Animals and was confirmed by the Ethics Review Committee for Animal Experimentation of Mazandaran University of Medical Sciences (IR.MAZUMS.REC.1398.1690). Then, the male Wistar rats (having 120 to 150 g weight) were anaesthetized (87 mg ketamine/kg of the body weight as well as 13 mg xylazine/kg). After that the electric hand razor was used to shave the abdominal skin rats. After 48 hours, chloroform inhalation was used to kill them. Then, removal of abdominal skin was done via surgery. The skin was cautiously cleansed from respective subcutaneous fat, and contacted with a saline solution for 24 hours prior to diffusion experiment. Next, the skin was placed in the Franz cells (with 3.8 cm^2^ diffusional area). Afterwards, the cut skin was put between the cell halves and dermis encountered with the receptor fluid. Later, the receiver part was filled with ethanol:water 80:20. In the next stage, the diffusion cells were kept at 32ºC (through thermo-statically controlled water circulated via a jacket around the cell body during tests). They were shaken at 150 rpm with the magnetic stirring bars during experiments. Therefore, receptor chamber temperature was generally adjusted at 32ºC for approximating normal skin conditions. 0.7 g (2800 μg Vit A) of Vit A-SLN gel (as a sample) and 0.7 g (2800 μg Vit A) of Vit A-suspension gel (as controls) was dispersed evenly on the shaved dorsal surfaces in the donor part sealed from the atmosphere via slow rubbing via a spatula. The specimens were extracted from receiver medium at the given periods (2,4, 6, 8, 10, and 24 hours) and the equal volume of the fresh alcohol:water 80:20 was added to the receiver phase. Each sample was purified through a syringe filter (pore size of 0.22 μm) and examined via HPLC technique as mentioned earlier. Hence, skin was discarded when the permeation examination ended. Then, deionized water was chosen to wash it 3 times. Then, it was dried for calculating the amounts of Vit A set down into the skin. Afterwards, the discarded skin was excised into the little pieces via a pair of scissors, transported to a tube and then digested in ethanol:water 80:20 for 24 hours. Afterwards, it was sonicated with bath sonicator for one hour. A filter paper was used to filter the supernatant. Next, it was filtered via a syringe filter (0.22 μm pore size). Then, HPLC was used to quantify it for determining the Vit A contents.^15^

### In vitro non-specific cytotoxicity

According to the research design, the *in vitro* experiments to analyze cytotoxicity of formulations was conducted with HFF human foreskinfibroblast cells as template. Then, the cells were seeded in the bottom micro-plates (Nunclon) with 96 wells and the density equal to 10^5^ cells/well with diverse doses of formulation, drug free SLN and free Vit A (10, 5, 2.5, 1 and 0.5 𝜇M) or vehicle control for 24 hours. Following the elimination of compounds, PBS was used to wash these cells and colorimetry formazan (MTT) was used to assess the cell viability. Notably, 3[4,5-di-methylthiazol-2-yl]-2,5 diphenyl-tetrazolium bromide (MTT) was considered a simplified reliable, and reproducible colorimetric procedure to gauge the mitochondrial metabolic decline of the yellow tetrazolium salt into insoluble formazan crystals in the aqueous solution of the viable cells. Then, incubation of the MTT (0.5 mg/mL) and cells was performed at 37°C for 4 hours. In the next stage, we removed supernatant and dissolved the formazan crystals in DMSO (100 µL). Moreover, agitation of these plates was performed for 20 minutes and optical density was gauged with a multi-wall spectrophotometer operation at 560 nm. The concentrations were tested three times with 6 other controls (the cells in medium). Finally, equation (2)^16^ was used to calculate the cells viability.

Viability (%)=[OD560(sample)/OD560 (control)]×100. Eq. (2)

### In vivo Biological Evaluation of Vit A-SLN

#### Animals

Male Wistar rats, weighing 120–150 g was utilized. They were housed six in each plastic cage in the animal room that had a temperature of 21±2°C on a 12 hours light/dark cycle (lights on 07:00–19:00 hours). Moreover, foodstuffs as well as water were provided at each time with the exception of the experimentation’s courses. We utilized each animal just one time. Notably, the ethical committee of Mazandaran University of Medical Sciences confirmed the study.

### Skin irritation test

As any research, we followed the protocol of the institutional animal ethics committee to do the tests. Therefore, hair on the dorsal side of the animals was removed by clipping one day prior to this section of experiment.^17^ Then, animals were divided into five groups (n=6) so that group I considered the control, group II was given Vit A-SLN (optimized formulation), group III was given the Vit A-suspension gel, group IV was taken the blank SLN gel and finally group V was taken 0.8% (vol/vol) aqueous solution of formalin as the standard irritant.^18^ A new formulation, placebo, Vit A-suspension gel, or the newly developed formalin solution was applied for three days every day. Ultimately, the used locations were constantly graded by the same author on the basis of a visual scoring scale.

### Statistical analyses

Data were represented as the mean ± standard deviation (SD). ANOVA, Tukey’s test, and Student t-test were used for comparing controls and the treated groups. Statistical analyses were done by SPSS2021. Finally, *P* value less than 0.05 was estimated as statistically significant.

## Results and Discussion

### Vit A-SLN characteristic analysis

As the size of NPs is one of the major features of NPs; thus, it has been specially considered. Table 1 reports hydrodynamic diameter of the NPs (the intensity weighted mean diameter or z-average diameter). PDI represents dispersion quality (as an indicator of the width of the particle size distribution).^19^ In general, PDI is in the range of 0-1 and PDI values greater than 0.7 shows a very wide-ranging distribution of the particle size.^20^ Table 1 illustrates NPs size and PDI can vary via changing the surfactants composition. In general, a more acceptable stability of emulsion droplets is ensured by using binary mixes of surfactants with small and great HLB values. Notably, the surfactants having low and high HLB may be distributed in the oily and aqueous phases, and result in higher stability of the surfactant films at the interfaces.^15^ It was found that each formulation with binary mixes of surfactants possesses proper particle sizes and Poly-dispersity index. This obviously indicated that adding co-emulsifier (Span®80) changes particle size in an irregular manner that might be a sign of more acceptable stability of the distributed formulation in the presence of another emulsifier.^19^ In other word, when HLB-value was set at 12 (near BW HLB), size of the particle of formulations considerably declined from 197.67 ± 22.942 nm to 64.85 ± 4.259 nm (*P*  <  0.05) (comparison of Vit A-SLN3 with other formulations in Table 1). All the SLN formulations via a binary mix of surfactants showed a PDI < 0.6, which indicates good distribution of SLNs. Even though the drug particles possess positive charge, ZP of various formulations has been negative ranging from -0.65 mV to -6.63 mV. Reports suggested that non-ionic surfactants unpredictably show a negative ZP surrounding colloidal particles that might result from the dipole nature of ethoxy groups of non-ionic surfactants.^20^ Moreover, as ratio of Tween: Span decreased from absolute, ZP of formulations enhanced from -2.87 ± 0.20 mV (Vit A-SLN1) to -6.63 ± 0.36 mV (Vit A-SLN5) (*P*  < 0.05), and particles achieved higher negative charges.

Results showed the enhanced tendency of the binary mixes of Tween 80 and Span 80 in comparison with the time a single surfactant has been applied; therefore, the binary surfactants may establish barriers with higher condensation surrounding the colloidal particles so that they provide particles with higher negative charges caused by more surfactants surrounding NPs (this issue has been presented earlier where non-ionic surfactants generate negative charges surrounding colloidal particles). However, it is not related to the formulation Vit A-SLN6, in which the amounts of Tween decrease compared to the amounts of Span in the formulation, indicating that Span to Tween ratio significantly affects zeta potential so that the highest ZP was achieved when these 2 surfactants ratio is near to each other. It is an attractive result because the ratio of both surfactants handles ZP and NPs size (Table 1). In general, more little NPs carry more surfactants surrounding it, and make NPs with higher negative changes. It does not relate to the condition when Vit A-SLN5 has been contrasted to Vit A-SLN3 regardless of the size. Apparently, the amounts of surfactants absorbed by the NPs may alter ZP.

In other words, the existence of the greater concentration Span lacks the film layer created by Tween, which causes a decline in ZP (see comparison of zeta potential of Vit A-SLN5 & Vit A-SLN6 in Table 1) due to less surfactants surrounding NPs. Electro-phoretic mobility of the particles decreases via SLNs surface coverage and declines ZP.^19,20^ Since the critical micelle concentration of Tween 80 is relatively low (about 0.015 mM), therefore, the concentration of Tween 80 monomers in the dispersion medium should be low.^21^ Such a situation shows that absorbing surfactant monomers over the lipids hydrophobic surface would be more probable instead of creating micelles. However, at the interface between the 2 immiscible liquids, including water and oil, orientation of the surfactants would happen with the respective hydrophobic group in the oil and the hydrophilic group in the water.^19^

In addition, additional decrease in the ratio of Tween 80:Span 80 to 0.289:1 in Vit A-SLN6 resulted in a decline in the ZP of SLNs from -6.63 ± 0.36 mV in Vit A-SLN5 to -0.65 ± 0.19 mV in Vit A-SLN6. The particles surface is fully covered by non-ionic surfactants, and using the increased concentration of the 2^nd^ surfactant; that is, Span possibly may degrade the coverage capability of the first surfactant completely.^19,20^

With regard to the effect of the binary surfactant’s ratio, it could be stated that using the 2^nd^ surfactant possibly at the increased concentration may break the surfactant films surrounding the particles and also declines zeta potential. Therefore, this research did not regard ZP value as one of the main parameters when selecting optimum formulation. The SLNs fully coated by non-ionic surfactants such as Tween 80 and Span 80 usually remained constant due to higher steric stabilization in spite of possessing a smaller ZP and lower electro-static stabilization.^20^

EE% measurements outputs indicated that EE% of various formulations ranged between 14.50 ± 2.681% to 66.01 ± 8.670%. However, miscibility and solubility of the drug in the lipid matrix as well as the lipid-phase polymorphic state might affect loading quantity. Maximum EE% is related to the formulations with the greatest HLB value of 12.08 with more little particle sizes.

Specific surface area enhances with a decline in the particle size. Therefore, drug EE% diminishes. As seen in Table 1, as particle size decreases to 64.85 ± 4.259, EE% increases by 66.01 ± 8.670%. Moreover, an inverse entrapment existed between the size of particle and EE% as reported by earlier research. Greater surface area enhances the drug tendency and mobility for escaping from matrix. Hence, like in more little particles, the total particle surfaces are greater and the drug repulsion increases. Thus, lower EE% would be observed.^22^

The recently developed technique, which was applied for preparing Vit A loaded nanoparticle is preferred over other reported method^23^ because it does not use any organic solvent, and NPs were combined into the gel base that might be the resulting product. Additionally, no research determined the effects of Span:Tween ratio required for optimizing Vit A-SLN to have a more acceptable function. The NPs carry drug much deeper into the hair follicles than conventional formulations. It was presented that NPs with diameter between 200 and 750 nm penetrate specially into the hair follicles.^24^ Among formulations, the optimized formulation (Vit A-SLN2) prepared via ultrasonication procedure possessed particle dimension of 275.00 ± 29.860 nm, ZP of -3.16 ± 0.44 mV, PDI of 0.321 ± 0.015, and drug entrapment of 61.30±6.700%.

Akbari et al provided the successful manufacturing of the SLNs loaded with naproxen with the probe ultra-sonication; therefore, it can be good carriers for local delivery of naproxen and skin. The findings revealed reducing HLB enhanced particle size, PDI, EE%, and ZP, reflecting the control of physio-chemical features of the SLNs for achieving the intended characteristics through HLB-value of the surfactants applied for manufacturing the SLNs.^15^

Kelidari et al produced and characterized SLNs consisting of spironolactone. Then, they evaluated such colloidal carriers potent for dermal administration. The SLNs were procured through a modified emulsion-solvent evaporation followed by ultrasonication. The particle size, ZP and EE% of spironolactone-loaded SLN were determined and were found to be between 88- 325 nm, negatively charged and the spironolactone EE% was less than 59%.^25^

Castro et al examined retinoic acid in SLNs. The researchers indicated remarkable modification of the EE% and particles dimension of the procured SLNs via altering the amounts of surfactant, type of surfactant, and HLB. An SLN particle size ranged between 227 and 667 nm. The EE% was estimated between 9.1%-93.5%.^26^

Jeon et al prepared and characterized SLNs consisting of Vit A palmitate. Then, they evaluated such colloidal carriers potent for dermal administration. The SLNs were procured through a modified hot melt method. The particle size, ZP and EE% of Vit A-loaded SLN were determined and were found 100 nm, negatively charged and the Vit A EE% was less than 99%. The concentration of Vit A in the mentioned article was 533.33 times less than the current study.^27^

Argimón et al examined Vit A palmitate in SLNs. The researchers indicated remarkable modification of the EE% and particle diameter of the procured SLNs via altering the amounts of surfactant and type of surfactant. The EE% was estimated 90%. The concentration of Vit A palmitate was 0.5% in the mentioned article. The palmitate derivate of Vit A is more lipophile than Vit A-acetate for this reason, EE% of the mentioned study is higher than the current study.^1^

### Rheological characterization of Vit A-SLN gel

**Figure 1:**
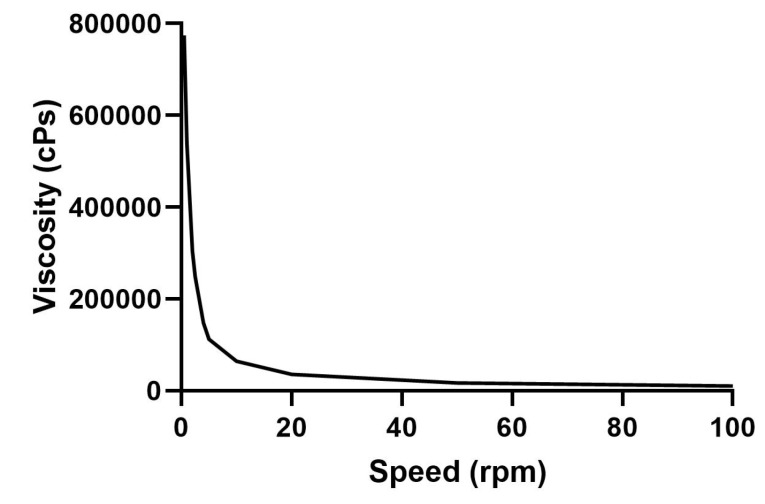
Viscosity profile of Vit A-SLN2 incorporating gel at room temperature

The rheological properties of lipid NPs have a profound impact on their ability for dermal use.^28^ Hence the rheological nature of gels prepared for topical drug delivery with Vit A-SLN was evaluated. The Vit A-SLN gels rheograms (Figure 1) displayed non-Newtonian flow patterns with no constant viscosity.^29^ The flow pattern of Vit A-SLN gels was also distinguished by shear-thinning property with variable thixotropy as the viscosity of the gel was reduced by increasing the shear rate.^30^ The merged shear thinning behavior and thixotropy are beneficial characteristics for topical formulations as they encourage flow and removal from the containers during application and enhance skin spreading. Moreover, the distributed film can rapidly obtain viscosity and therefore avoid running.^14^

### TEM analysis

**Figure 2:**
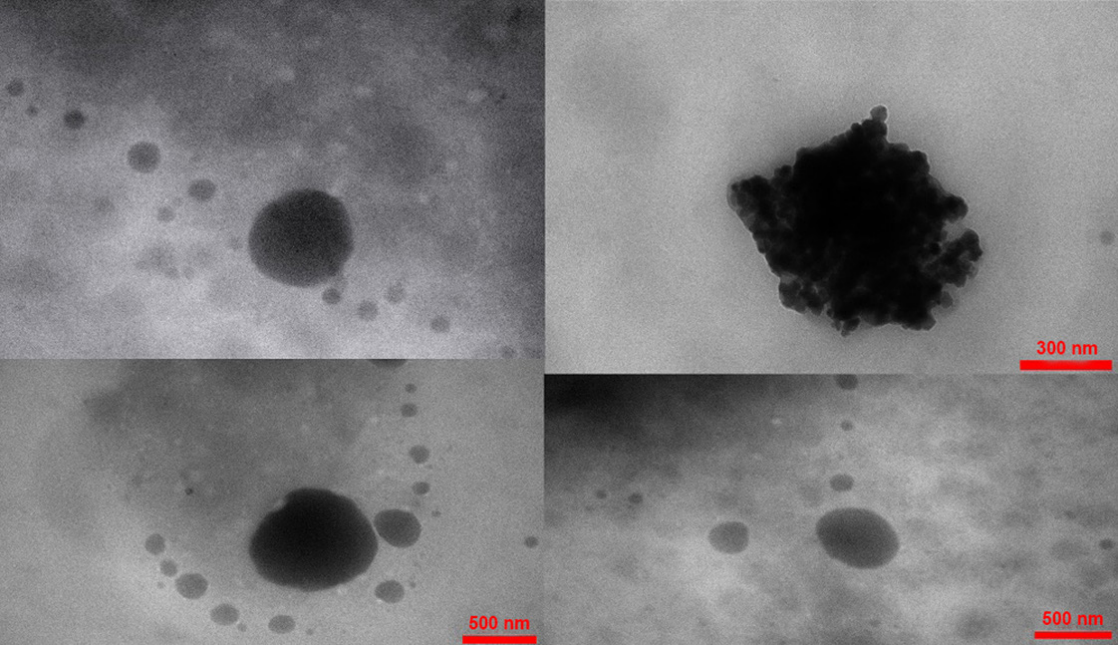
Various TEM micrographs of Vit A-solid lipid nanoparticles (Vit A-SLN2)

For showing microscopy images of NPs, Vit A-SLN2 was chosen as an optimized formulation. Figure 2 illustrates its microscope image. The image demonstrates segregation of particles, uniform sizes, and spherical shapes.

### ATR-FTIR spectroscopic analyses

**Figure 3:**
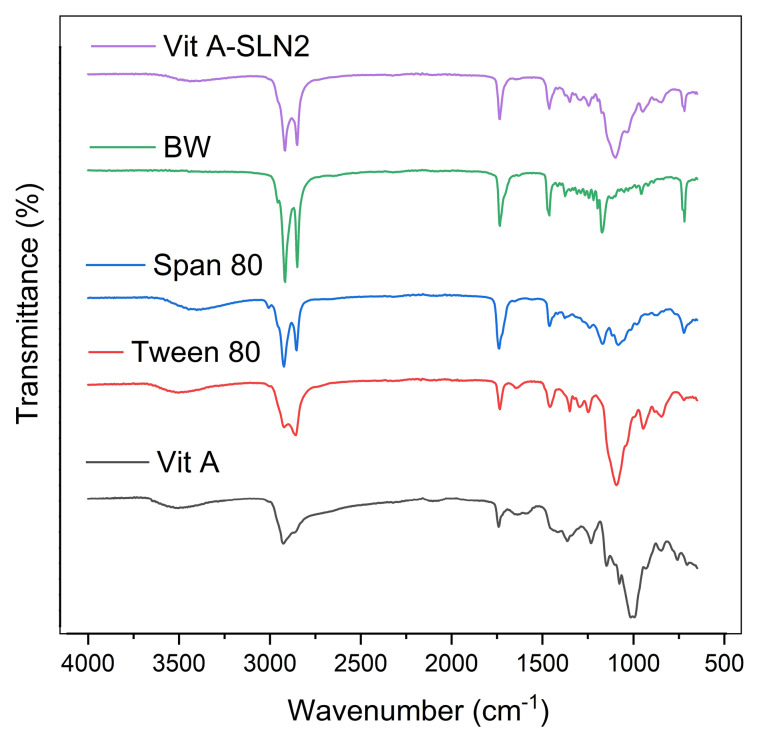
ATR-FTIR spectra of Vit A acetate, Tween80, Span80, BW, and Vit A-SLN2

Figure 3 illustrated ATR-FTIR of Vit A acetate, Tween 80, Span 80, BW, and Vit A-SLN2. Characteristic peaks of Vit A acetate were observed in the ATR-FTIRspectra of Vit A-SLN2 sample. According to results of ATR-FTIR, any chemical interaction was not found between the drug and excipients. The result of ATR-FTIR spectra of Vit A acetate, Tween 80, Span 80, and BW were summarized as follows:

Vit A acetate: 2925 cm^-1^ (C-H stretching) and 1740 cm^-1^ (C=O stretching); Tween 80: 3497 cm^-1^ (O-H stretching), 2922 cm^-1^ (-CH_2_- asymmetric stretching), 2858 cm^-1^ (-CH_2_- symmetric stretching) and 1735 cm^-1^(C=O stretching); Span 80: 3400 cm^-1^(O-H stretching), 2923 cm^-1^ (-CH_2_- asymmetric stretching), 2854 cm^-1^ (-CH_2_- symmetric stretching) and 1739 cm^-1^(C=O stretching); BW: 2917 cm^-1^(-CH_2_- asymmetric stretching), 2849 cm^-1^(-CH_2_- symmetric stretching), 1734 cm^-1^(C=O stretching), 1463 cm^-1^(-CH_2_- bending) and 1171 cm^-1^ (C-O stretching).

### DSC analysis

**Figure 4:**
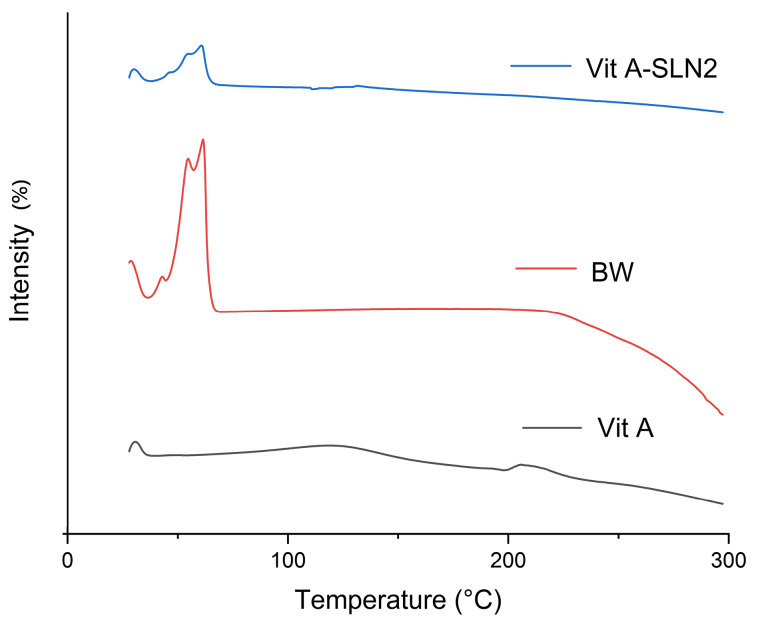
DSC traces of Vit A, BW, and Vit A-SLN2

Thermal behavior of Vit A, BW, and Vit A-SLN2 powder was examined through DSC. Figure 4 shows DSC thermo-grams. Content of moisture of Vit A powder was removed at 105°C.^31^ The melting point was seen at 210°C. BW exhibited an endothermic peak ~40 to 66°C relative to the respective melting point.^15^ Moreover, the DSC thermo-grams of the examined SLN formulation included just endothermic peak surrounding the BW melting point. However, endothermic peak of the Vit A disappeared (Figure 4), indicating molecular dispersion of Vit A in SLN within the SLN.

### PXRD analyses

**Figure 5:**
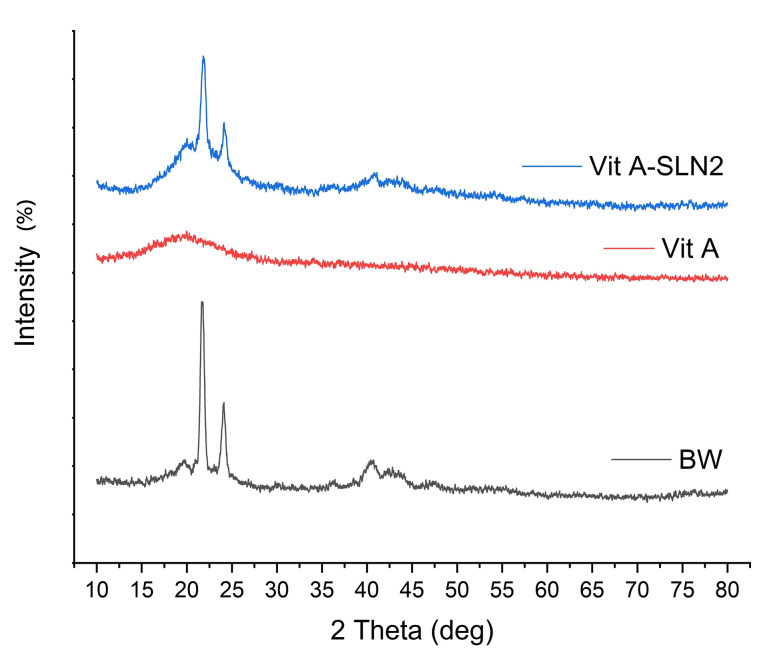
XRD of BW, Vit A and Vit A-SLN2

In this regard, Figure 5 explains the powder X ray diffraction (PXRD) patterns of BW, Vit A, and Vit A-SLN2. A wide spectral peak of the RA centered at 2θ=13° indicates a semi-crystalline structure.^31^ The BW indicated the peaks at 2θ:19.553◦, 21.656◦, 24.025◦, 30.12◦, 36.252◦, 40.45◦, 47.33◦, 55.13◦, showing the crystalline nature of BW.^20^ PXRD pattern of Vit A-SLN2 exhibited the peaks at 2θ: 21.384◦, 23.717◦, 40.23◦, 42.97◦, and 48.34◦. According to Figure 5, lipid peaks did not change; however, they experienced a declined intensity in comparison with the free lipid. Incorporating Vit A between the BW parts resulting in changes in the lipid crystallinity may involve in creating the above condition. Findings represented an acceptable entrapment of Vit A in BW with no interactions that are in line with DSC and ATR-FTIRanalyses.

### In vitro skin absorption examination

**Figure 6:**
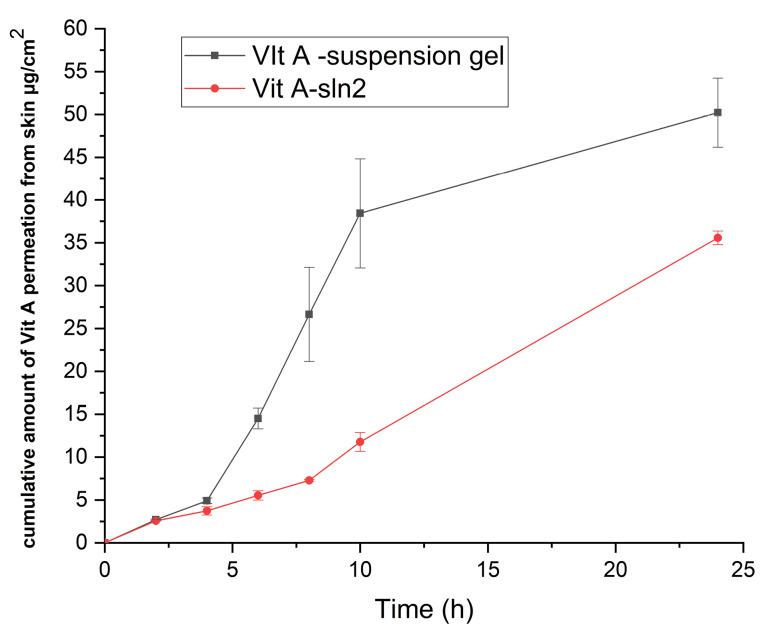
The cumulative amount of Vit A permeated across rat skin (data are mean and standard deviation of three determinations, n=3;ANOVA test followed by Tukey’s test showed that the effect of time and formulation on Vit A permeation was significant *P* < 0.05.) Sample represents Vit A-SLN2 gel and standard is Vit A suspension gel containing the same concentration of Vit A

**Figure 7:**
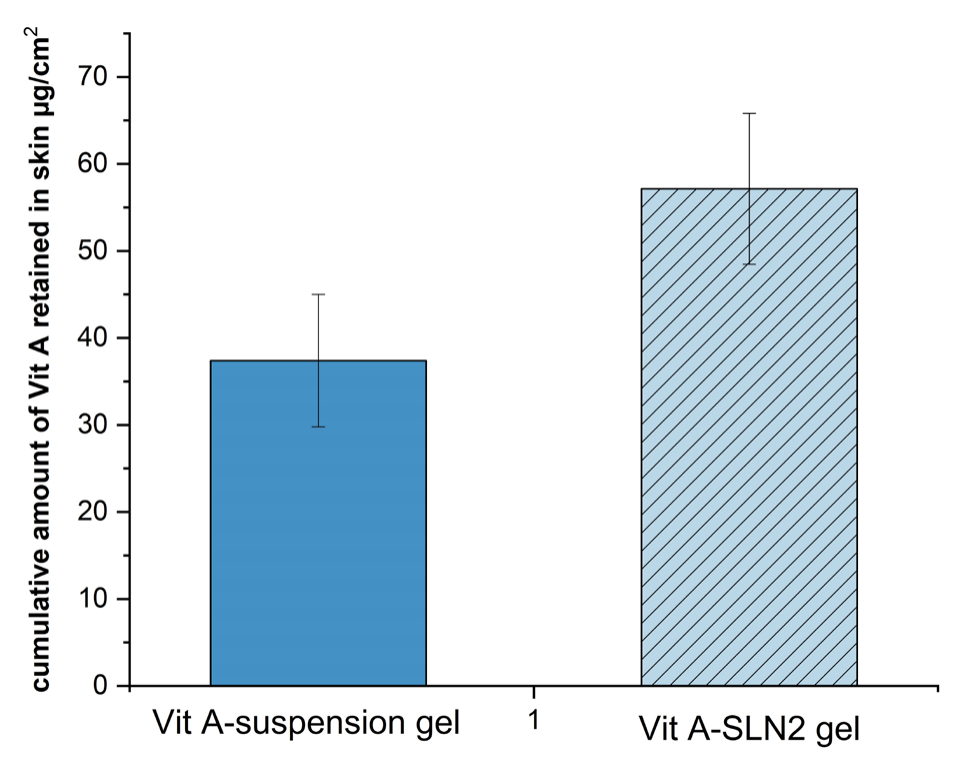
Amount of Vit A penetrating to the skin (3.8 cm^2^)layers after 24 h (Dermal delivery) (error bars are standard deviation, sample represents Vit A-SLN2 and standard formulation is the Vit A suspension gel containing the same concentration of Vit A; t-test was carried out between the sample and standard. The difference was significant *P* < 0.05; n=3)

According to the research design, the rats’ skins were applied in examining per-cutaneous absorption. Even though extensive reports showed that the human skin is capable of providing more reliable skin absorption data in comparison with the rat skin,^32,33^ these studies often cannot be applied specially during the early evolution of a newly developed drug formulation. Additionally, there is a general census that the human skin has lower permeability compared to the rat’s.^34^ Nevertheless, since the purpose of the present research has been determining performance of the formulation provided with the functions of the earlier research, in which the rat skin model was applied, researchers employed the rat skin model for allowing for a comparison. Therefore, Figures 6 and 7 demonstrate the cumulative plots of amounts of Vit A permeated via the rats’ skins (transdermal delivery) as a function of time and the amounts of Vit A permeated to the skin layers (dermal delivery). For evaluating the skin, which targeted potential of SLNs, permeability of Vit A within and via the skin was assessed by Franz diffusion cells. Moreover, in-vitro percutaneous absorption was conducted for assessing Vit A-SLN2 nanogel performance and comparing it with the suspension Vit A-gel performance. This experiment deal with Vit A-SLN capability for penetrating various layers of the skins and the skins penetration. Figures 6 and 7 show outputs. Nano-gel experienced an elevated penetration into skin layers (*P*  < 0.05), indicating that the newly developed Vit A-SLN gel formulation causes more acceptable local effects in comparison with Vit A suspension gel. It is found that more antiaging effects might be predicted for the drug loaded SLN gel in comparison with the plain drug gel. The amounts of Vit A measured in the receptor chamber for Vit A suspension gel was greater compared to the when Vit A-SLN2 gel was applied (*P*  <  0.05).

The above result demonstrates the feature of Vit A-SLN2 gel formulation for decreasing systemic uptake and consequences and improving the levels of the drug obtained at the skin location. Also, the polymer can protect the drug from the physiological environment by improving its stability. Moreover, the gelling properties of carbopol polymers are also very important for drug permeation enhancement from formulations.^35^

Lai et al observed possible improvement of accumulating the oil of the formulations in skins using SLN, whereas regarding the control solution (traditional formulation), the oil leakage was observed via skins (lower oil in layers of the skins).^36^ In this regard, Puglia et al revealed the particles can decline the drug penetration; however, they can increase the leakage and accumulation in the horny layer at the same time.^37^

Moreover, Madan et al demonstrated that the skins deposition of mometasone from the gels with SLNs is 2.67 times greater than the marketed cream as well as 20 times greater than the plain medicine loaded gel.^38^

In addition, Bhalekar et al revealed that miconazole nitrate –SLN gel can considerably enhance accumulative uptakes of the miconazole nitrate into skins on the marketed gel with a remarkably increased the skins’ targeting impact.^39^ Outputs of in-vitro percutaneous absorption in this research showed that lipid NPs gel would be hopeful in local delivery for Vit A and stimulation of novel and deeper examinations in this area.

### In vitro cell viability study

It is widely accepted that Vit A contains a group of not less than 50 structurally associated chemical compounds. On the one hand, the word “Retinoids” was presented in 1976 for describing the retinol (Vit A) and the respective synthetic and natural derivatives.^40^ For example, retinyl acetate and retinyl palmitate are considered as the forms of retinoids with the greatest applications in the cosmetics. It is notable that following the utilization on the skins surfaces with medical or cosmetic ointments, retinyl esters were kept in the keratinocytes wherein they were metabolized into the biologically-active retinoids.^40^ Moreover, retinoids mediated their biological effects via binding to the nuclear receptors.

Actually, retinoids in the skins contributed to differentiate the keratinocyte cells, reduced the epidermis cells adhesion, and finally facilitated the corneocyte exfoliation from the epidermis surfaces. However, the retinoids in dermis regulated the fibro-blast proliferation or rapid growth, induced angiogenesis, and played crucial roles in synthesizing the elastin fibrils and collagen. It is notable that since the retinoids are capable of inhibiting the transformation of arachidonic acid, they possessed anti-inflammatory features too. Moreover, the retinoids regulated melanogenesis and contributed to the distribution of the melanin granules to keratinocytes. Finally, as the retinoids normalized the exfoliation procedures of the sebaceous glands, they had anti-comedogenic features too.^40,41^

**Figure 8:**
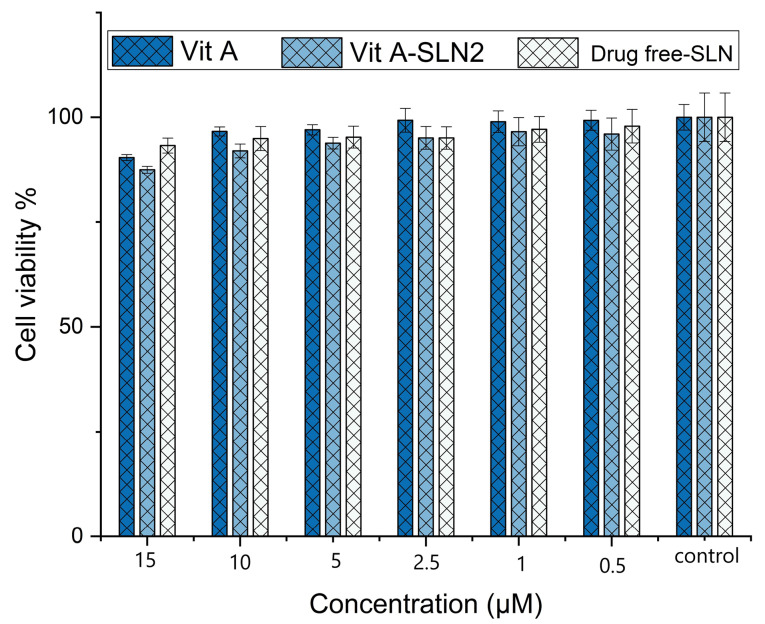
Cell viability of drug-free SLNs and different concentrations of Vit A-SLNs. Data are expressed as mean ± SD

Therefore, in order to assess the model usability, well-known techniques to experiment the nano formulated medicines were utilized as compared in the study. In addition, MTT assay is considered as one of the absorbance-based experiments with the ability of the measurement of the metabolic activities of the living cells with a frequent utilization as a result of their simple operation.^42^ This needs the extended experimental interval since 24 hours or longer treatments has been commonly required for experiments.^43^ In order to analyze the cell viability capability of Vit A-SLN2, different concentrations of free Vit A, drug free SLN and Vit A-loaded SLN at 0.5-15 µM were incubated with HFF normal fibroblast cell line obtaining from Pastor Institute (Tehran, Iran) for 24 hours (Figure 8). In equal concentrations of 0.5-10 µM, there was no significant reduction in the viability of cells over 24 h hours in the presence of Vit A, blank SLN and Vit A-SLN2 *(P*> 0.05*).* After treatment with 15 µM of blank SLN and Vit A-SLN2 for 24 hours there was significant reduction in the viability of cells over 24 hours, 93 and 87 % of cells survived, respectively; while this percentage was same for the Vit A *(P < *0.05). The IC50 of Vit A-SLN2 and Vit A was 6039 M and 268 µM, respectively.

In this regard, Zhang et al^44^ illustrated time- and dose-dependent cytotoxic impacts of ATRA mediated by dysfunctioning the mitochondria, changing the cell cycles, and inducing apoptosis. Moreover, process of the retinoic acid induced apoptosis depended on Bcl-2 protein,^45^ nuclear transcription factor TR3, Bax,^46^ transcription factor Ets1,^47^ activation of p38, as well as the caspase cascade, but not via p53.^48^

Therapeutic nanodrugs may cause reduction in the viability of cells. Moreover, the little particles experienced simple transfection to the cells and caused decrease cell viability. In addition, surface charge of the NPs increased reactivity with the cells and proteins. Furthermore, the free oxygen radicals are so reactive that resulted in the oxidative stress. Since the drug or gene has been loaded on the NPs, treatment activities must be specified and contrasted with the free drug for specifying the effects of encapsulation.^49^

### Skin irritation test

The real value of the delivery system was explained with regard to its capability for delivering the efficient concentration of active agents with no threats to the safety dimensions. Put differently, it is necessary to release medicine from the delivery system so that it generated no irritation, cyto-toxicity, or immunogenicity. Focusing on the dermatological applications, this system must avoid dermal sensitization reaction in case of the occurrence with the active agents.^50^ Considering the dermal topical preparation, the skin irritation was a consequence with common encounters; nonetheless, the skin irritation could have a direct correlation with the active agent concentration. Therefore, this delivery system that was loaded with an active agent could essentially contributed via modulation of its release and thus could alter this association via facilitation of the intra-follicular permeation and decline of its per-cutaneous uptake.^51^

A number of investigators controlled the skin irritation potential of the SLNs-based topical formulation with the evaluation of the outputs for edema and erythema that utilized the rats as the animal models.

Testing the skin irritation of the topical formulation of Vit A-SLN2 demonstrated a skin irritation score (edema and erythema) of < 2 (Table 2). With regard to Draize, Woodward, and Calvery, those compounds generating the scores of ≤2 are regarded as negative (without skin irritation). Therefore, Vit A-SLN gel formulation did not exhibit any irritation and enhanced the patient suitability and skin acceptability. Moreover, and it is safe for the topical utilizations.

**Table 2:** Skin Irritation Scores Following dermal application

**Rat num**	**Control**		**Vit A-SLN2 gel**		**Vit A Suspension gel**		**Blank SLN gel**		**Formalin**	
	**Erythema** ^a^	**Edema** ^b^	**Erythema** ^a^	**Edema** ^b^	**Erythem** ^a^ **a**	**Edema** ^b^	**Erythema** ^a^	**Edema** ^b^	**Erythema** ^a^	**Edema** ^b^
1	0	0	1	0	2	1	1	1	3	3
2	0	0	0	0	1	2	0	0	3	3
3	0	0	0	0	1	2	1	1	4	3
4	0	0	0	1	2	1	1	1	3	3
5	0	0	0	0	1	1	1	0	4	3
6	0	0	1	1	2	2	1	2	3	3
Score			0.33±0.51*	0.33±0.51*	1.50±0.54*	1.50±0.54*	0.83±0.40*	0.83±0.75*	3.33±0.51	3.00±0.00

^a^Erythema scale: 0, none; 1, slight; 2, well defined; 3, moderate; and 4, scar formation. ^b^Edema scale: 0, none; 1, slight; 2, well defined; 3, moderate; and 4, severe. *Significant compared with formalin (*P* < 0.05).

The crucial barrier to utilize the topical retinoids is considered to be the increased incidence of the skin irritation. In fact, it is possible that patients possibly enhance dermatitis with the skin tenderness and redness. Nonetheless, patients discontinued treatments due to such reactions.^52^ NPs were used to overcome this complication. The SLNs are the novel colloidal delivery systems, offering encouraging tools to deliver the drug, especially for the poorly water-soluble medicines. These NPs offered several dermatological and cosmetic characteristics like the skin adhesive features, occlusion, and greater skin hydration. The small sizes of the lipid particles ensured the close contacts to the stratum corneum and could enhance the amounts of the drugs permeating into the skin or mucosa. Because of their solid lipid matrix, the ground for the controlled release from the carriers is provided, which is a vital means for supplying the drugs over a longer duration for the reduction of the systemic absorption and when drugs are irritating at the increased concentration.^53,54^

Golmohammadzadeh et al showed that isotretinoin-SLNs formulations considerably less irritated in comparison with the commercial isotretinoin-GEL that showed its potent to the improvement of the skin tolerability and one of the carriers for the topical delivery of isotretinoin.^55^ Also May El-Samahy et al showed that for treating the facial acne vulgaris nanosomal retinol is more efficacious and better tolerated than its conventional formulation with nearly no side effects and no irritation for use.^56^ Finally, Shrotriya et al indicated that repetitive use of resveratrol-SLN gel did not show any skin irritancy (score equal to 0) on the rabbits’ skins. Therefore, resveratrol-SLN gel formulation did not exhibit any irritation and improved the patient suitability and skin acceptability. Finally, it is safer for topical applications.^57^

## Conclusion

This research recommended that SLNs would be proper carriers for skin delivery of Vit A. This carrier may enhance dermal delivery of Vit A via a greater concentration into the skin deeper layers. For achieving NPs with higher stability and localizing Vit A delivery, the solid lipid nano-particle formulations should be optimized. Studies indicate that Span:Tween ratio is a vital factor for optimizing Vit A nano-gel formulation to obtain an acceptable delivery to the skin. Thus, *in vitro* cytotoxicity studies revealed nontoxicity of the optimized Vit A-loaded SLNs because of the greater percentage of the cell viability equal to 84%. Finally, the presented approach can be considered as one of the suitable vehicles for dermal delivery of Vit A. Skin irritation test proved the non-irritancy of the applied SLN gel components.

## Ethical Issues

All animal studies were approved by the Ethics Review Committee for Animal Experimentation of Mazandaran University of medical sciences under registration ≠ ir.mazums.rec.95.2471. All animal experiments were in accordance with ARRIVE guidelines and were carried out in accordance with the U.K. Animals (Scientific Procedures) Act 1986 and associated guidelines, EU Directive 2010/63/EU for animal experiments.

## Conflicts of Interest

The authors declare no conflict of interest.

## Acknowledgments

The present research has been financially supported by the grant awarded by the investigation council of Mazandaran University of Medical Sciences.
